# Congenital insensitivity to pain with anhydrosis: report of a family case

**DOI:** 10.4314/pamj.v9i1.71209

**Published:** 2011-07-25

**Authors:** Smael Labib, Mohamed Adnane Berdai, Sanae Abourazzak, Mustapha Hida, Mustapha Harandou

**Affiliations:** 1 Department of anesthesia and intensive care, University Hospital Hassan II, Fez, Morocco; 2 Department of Pediatrics, University Hospital Hassan II, Fez, Morocco

**Keywords:** Congenital insensitivity to pain, hereditary, neuropathy, sensory and autonomic neuropathy, family case, anesthesia

## Abstract

Congenital Insensitivity to pain with anhydrosis (CIPA) is a rare inherited disease. It is classified as hereditary sensory and autonomic neuropathy type IV. Pain insensitivity and autonomic deficits are present, but touch and pressure sensitivity are unimpaired. Mental retardation is usually present. We report a family case of a 5 years old girl and 2 years old boy with congenital insensitivity to pain, while discussing the clinical features and the anesthetic strategy of such patients. Patients with Congenital Insensitivity to Pain with anhydrosis may undergo surgery because of susceptibility to trauma due to absence of pain. The clinical features may intrinsically possess anesthetic challenges.

## Introduction

Congenital insensitivity to pain with anhydrosis (CIPA) or hereditary sensory and autonomic neuropathy type IV (HSAN IV) is a rare autosomal recessive neuropathy of the group of hereditary sensory and autonomic neuropathies (HSAN), first described in 1932 by Dearborn as “Congenital pure analgesia” [1] , and an exceedingly rare clinical disorder characterized by insensitivity to pain with intact tactile perception ,self mutilation, recurrent unexplained fever, anhydrosis, mental retardation and autonomic nervous system abnormality since infancy. Patients with CIPA often experience trauma, bony fractures, and osteomyelitis because of insensitivity to pain. Therefore, such patients may undergo surgery such as osteotomy and amputation. Since it is a rare condition, reports on the anesthetic conduct in patients with CIPA are not easily found in the literature. The objective of this report was to present family cases with CIPA, and to discuss the anesthetic strategy of such patients.

## Case report

Written informed consent was obtained from the patient for publication of this case report and accompanying images. A 5 years old girl and a 2 years old boy, siblings, were born after a normal pregnancy and normal delivery. They were the only children of a healthy family and of a non-consanguineous marriage. There were no family history of neurologic or metabolic disorders and no other member of their family was diagnosed with CIPA.

They were brought by their parents looking for a therapy that could avoid injuries to their oral structures and the finger biting. Their physician had suggested full mouth extraction, but the parents did not agree because of the psychological and functional implications.

Their parents relate a history of self-mutilation from first months of life. After their teeth started to grow in, they began to chew their fingers and bit off the tip of their tongue, since eruption of the teeth there had been biting of their tongue, fingers, wrists, and feet, causing bleeding. They exhibited an absence of normal reaction to painful stimuli such as falls, cuts, and injections (e.g.: for vaccinations).

**Figure 1:**
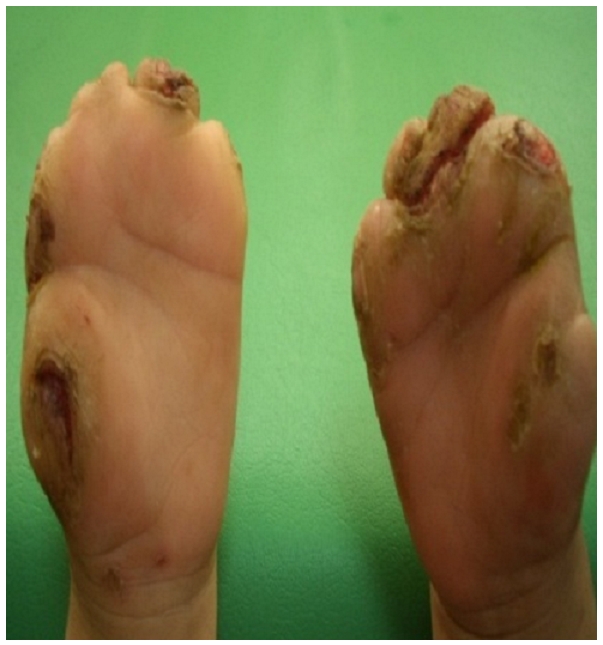
Self-mutilation of interphalangeal joints of fingers in a child suffering from congenital insensitivity to pain with anhydrosis

**Figure 2:**
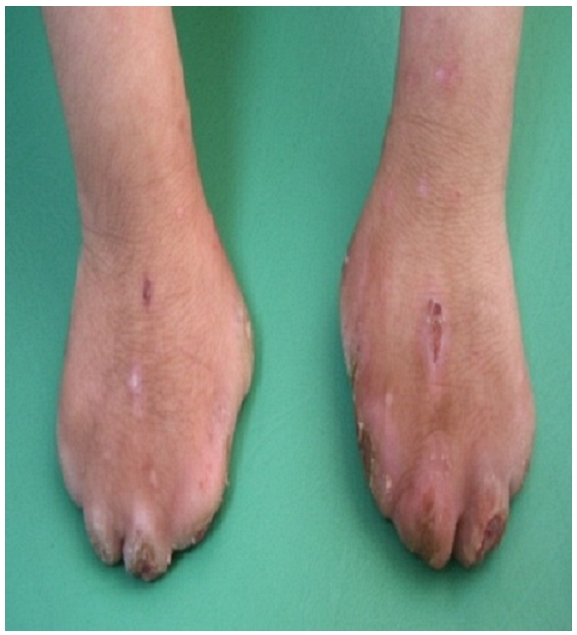
Self-mutilation of interphalangeal joints of toes in a child suffering from congenital insensitivity to pain with anhydrosis

On admission, they were poorly developed and thin, their tongue and upper lip had been bitten off. They had scars from injuries and they had self-mutilation of interphalangeal joints of the fingers and toes (Figure 1 and Figure 2). The distal thumb and the tips of two fingers on the right hand and the toes were missing. Neurologic examination revealed depressed deep tendon reflexes and decreased tactile sensitivity. The patients seemed to respond appropriately to thermal stimuli. However, there was no reaction to painful stimuli. They showed normal lacrimation, salivation and corneal reflex, presence of a significant loss of tissue at the soles of the feet, toes and fingers. It was noted that their general development had continued normally, and they were of normal intelligence, the only difference in clinical presentation of the two siblings is that the girl had chronic diarrhea, probably related to autonomic dysfunction, with sphincter incontinence.

**Figure 3:**
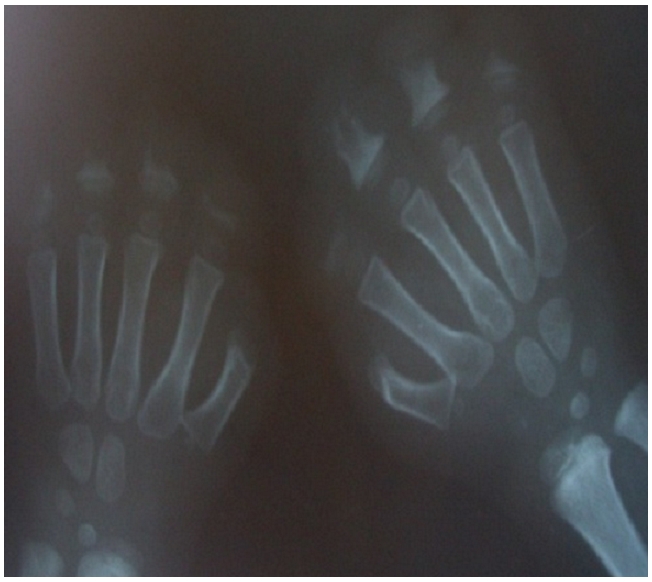
X-ray showing destruction and amputation of fingers in a child suffering from congenital insensitivity to pain with anhydrosis

Paraclinical investigations were common to both patients. Laboratory examination including white blood cell, hemoglobin, serum electrolytes, GOT, GPT, renal function value, uric acid, creatine kinase, were normal. Hands and feet radiograph showed destruction and amputation of fingers and toes (Figure 3).

Once the diagnosis was confirmed, the patients benefited from a multidisciplinary management. Self-mutilation of the tongue, lips and fingers had posed difficult problems since the dental eruption; stomatologists tried to make a mouth guard, they estimate that extraction of all primary teeth was a radical alternative that should be considered only if all other possibilities failed or in extreme cases. Psychological support to the patients and the family were also necessary. Genetic counseling was indispensable given the seriousness of the disease. Parents were also advised to consult in cases of fever to eliminate a potential infection and in case of injury to detect an unnoticed fracture.

## Discussion

There are number of diseases that affect sensory function from birth. Dick [2] classified these congenital neuropathies into five types: sensory radicular neuropathy, congenital sensory neuropathy, familial dysautonomia or Riley-Day syndrome, congenital insensitivity to pain with anhydrosis (CIPA), and congenital indifference to pain. The differential diagnosis of these neuropathies is not straightforward because the clinical pictures overlap.

CIPA is characterized by insensitivity to superficial and deep painful stimuli with intact tactile perception, self-mutilation, recurrent unexplained fever in infancy, anhydrosis, mental retardation, absent or hypoactive deep tendon reflex, normal corneal reflex and lacrimation and autonomic nervous system abnormality.

Autonomic abnormalities include the inability to sweat in response to heat or chemical stimuli (pilocarpine) and the production of a wheal but not a flare after intradermal histamine injection [3]. Individuals with HSAN IV show an absence of unmyelinated fibers and losses of small myelinated fibers [4]. Skin biopsy show an absence of epidermal innervation and loss of most dermal innervations as well as accompanying loss of unmyelinated and thinly myelinated fibers from the sural nerve, sweat glands show no innervation [5]. The condition is caused by autosomal recessive mutations and polymorphisms in the TRKA gene on chromosome 1 that encodes the receptor tyrosine kinase for nerve growth factor (NGF) [6]. Patients with CIPA often experience trauma, bony fractures, and osteomyelitis because of insensitivity to pain. Therefore, such patients may undergo surgery such as osteotomy and amputation.

It may be thought that patients with CIPA do not need anesthesia to undergo surgical procedures because of the insensitivity to pain. However, the tactile sensation is intact and some patients have tactile hyperesthesia [7]. Some patients have complained of pain in the postoperative period [8]. Therefore, surgical stimulation may produce unpleasant sensations. Thus, it is possible that the use of anesthetics can prevent undue tactile discomfort [7]. Also, anesthetics are useful to ensure cooperation and immobility during surgery for a pediatric patient with behavioral problems.

Some authors described the difference in concentration of inhaled anesthetics needed for orthopedic surgery and dental surgery. According to their report, the inhaled anesthetic was≥1 MAC for orthopedic surgery, and≤1.0 minimum alveolar concentration for dental surgery. They speculated that these differences were caused by the nociceptive stimulus of each operation. However, there have been few reports concerning the anesthetic management of patients with CIPA because it is a rare disease [8,9]. Patients with CIPA have autonomic and nociceptive dysfunction; therefore, the anesthetic conduct represents a challenge for the anesthesiologist.

Congenital hyposensitivity to pain is a rare disease, and there is limited information regarding anesthetic treatment of these patients. Okuda et al [8] reported 6 patients with HSAN IV who underwent 20 operations during general anesthesia without any adverse events. All patients received standard anesthesia management including inhalational agents in doses that are expected to be administered to patients with normal sensitivity to pain. No patient received opioids during operations, and no perioperative complications were noted.

Tomioka et al [7] reported that a variety of pre-anesthetic medications were given to the patients at the various medical facilities. In 19 of the 45 cases, atropine was given as a pre-anesthetic medication; despite the recommendations that avoid anticholinergic drugs because of the risk of hyperthermia [10], however, none of the patients displayed signs of heat. In 19 cases, sedatives such as a benzodiazepine or hydroxyzine, and meperidine were given as a preanesthetic medication. These medications all had a normal effect on the patients. All of the procedures were performed under general anesthesia. They reviewed 15 patients with HSAN IV who had 45 operations during general anesthesia using different inhalational agents (Halothane, Enflurane, and Sevoflurane) within wide range of anesthetic concentrations and intra venous anesthetic included barbiturates, benzodiazepine, propofol, and ketamine [7]. Four patients received opioids intraoperatively, and two received fentanyl-supplemented inhalational.

The primary anesthetic concern in patients with HSAN type IV is impaired temperature control, which can cause death in about 20% of the affected patients within the first 3 years of life [4]. Strict perioperative temperature control is recommended to maintain body temperature at about 37 °C [11,12]. The elevation of the temperature can be prevented by adequate monitoring, adjusting the temperature of the operating room, and using thermal mattresses. However, there is no association between malignant hyperpyrexia and CIPA. The genetic mechanisms of precipitating hyperpyrexia in the 2 conditions, malignant hyperpyrexia and CIPA, are different and previous reports do not reveal any episodes of malignant hyperpyrexia in these patients after exposure to suxamethonium and inhalational agents, including halothane, enflurane, isoflurane, and sevoflurane [4,7, 9,12].

There are reports of surgical procedures without anesthesia in patients with CIPA, such as the case of a patient who underwent amputation of both feet under sedation (20mg midazolam and thiopental over the 3 hours, but without analgesia (opioids) or general anesthesia. He did not show any response to incision of the skin or disarticulation, and he only reacted to clamping of a nerve trunk with flexion of a limb [13]. An eight-year old patient with CIPA underwent reduction of a femur fracture with osteosynthesis under epidural block and sedation without complications during the procedure [14].

## Conclusion

Congenital insensitivity to pain with anhydrosis (CIPA) or hereditary sensory and autonomic neuropathy type IV (HSAN IV) is a rare autosomal recessive neuropathy of the group of hereditary sensory and autonomic neuropathies (HSAN). Patients with CIPA may undergo surgery because of susceptibility to trauma due to absence of pain. The clinical features may intrinsically possess anesthetic challenges.
